# Human rights and COVID-19 triage: a comment on the Bath protocol

**DOI:** 10.1136/medethics-2021-107361

**Published:** 2021-04-16

**Authors:** Vivek Bhatt, Sabine Michalowski, Aaron Wyllie, Margot Kuylen, Wayne Martin

**Affiliations:** 1 Essex Autonomy Project, University of Essex, Colchester, UK; 2 Human Rights Centre, University of Essex, Colchester, Essex, UK; 3 School of Law, University of Essex, Colchester, Essex, UK; 4 School of Health and Social Care, University of Essex, Colchester, Essex, UK

**Keywords:** COVID-19, ethics, rights, ethics committees/consultation, resource allocation

## Abstract

In their discussion paper of November 2020, Cook *et al* present a draft protocol for navigating circumstances in which emergency services are overwhelmed. Their paper suggests that COVID-related triage decisions should be based on clinical assessment, patient and family consultation, and a range of ethical considerations. In this response, we note that the protocol exhibits an ambiguity that is likely to result in irresolvable dilemmas when put into practice. This ambiguity is exemplified in the paper’s prime ethical imperative (to ‘save more lives and more years of life’), which takes the form of an undefined conjunction whose practical implications are left unspecified. We see this ambiguity in the prime imperative as one manifestation of a broader set of tensions in the protocol. We show that the discipline of human rights provides an essential supplement to the ethical framework on which Cook and colleagues rely, providing a framework for understanding and working through triage dilemmas involving age, discrimination and equality.

## Introduction

In the absence of a national COVID-19 triage policy, local hospital ethics committees across the NHS have had to develop their own policies and procedures for navigating circumstances in which emergency services are overwhelmed. In the context of these challenging deliberations, the discussion paper and draft triage protocol produced by Cook and colleagues at the Royal United Hospital Bath is a welcome contribution.^1^ On many particular points, we find ourselves sympathetic to what we shall refer to as ‘the Bath protocol.’ However, we note that the proposed protocol exhibits an ambiguity and can be expected to yield practical dilemmas when put into practice. These dilemmas resist resolution within the proposed framework and point towards the need for a more direct incorporation of principles drawn from human rights.

One of the many welcome features of the Bath discussion paper is its attempt to be explicit about the ethical underpinnings of the approach it recommends. The main body of the discussion paper articulates a set of eight ‘ethical factors’—factors which are elaborated in an online Appendix devoted to ‘ethical context in a pandemic.’ The first and pre-eminent ethical consideration underlying the Bath protocol is articulated as follows: ‘Aim to save more lives and more years of life.’ It is notable that this prime imperative of the Bath protocol takes the form of a conjunction. Save more *lives* AND save more *years of life*. The two conjuncts in this imperative are not equivalent, but neither the protocol nor its ethical appendix clarifies the relationship between them. This ambiguity creates the potential for practical dilemmas in circumstances where the two conjuncts pull in different directions.

We see this ambiguity in the prime imperative as one manifestation of a broader set of tensions in the protocol pertaining to age, discrimination and equality. For example, the protocol firmly rejects the use of age as a sole determinant of ICU admission, claiming that such a policy would be discriminatory. But at the same time, it recognises that applying the protocol “may mean giving priority to younger patients.” Once again, neither the protocol itself nor the ethical appendix explains the basis for rejecting an age-based policy while endorsing one based on life-years. We suggest that these gaps and tensions in the Bath protocol can be traced back to a common source: the absence of sufficiently robust human rights considerations from its ethical substructure. Attention to human rights principles is of course a legal obligation. We believe that it also holds the key for improving the Bath protocol by (1) more clearly articulating the ethical foundations on which it is built, (2) providing a principled framework for resolving the ambiguity in its first principle and (3) raising a warning flag regarding one of its most controversial features.

## Discrimination

Decisions regarding the allocation of critical care resources clearly engage the right to life, which is protected under international,^2^ European^3^ and domestic human rights law.^4^ But measures undertaken to protect life are also subject to other important human rights considerations, principally the prohibition of discrimination.^5 6^ A discriminatory policy or practice is one that directly or indirectly disadvantages members of certain groups, such as older people, without an objective and reasonable justification for their differential treatment. This is a first area where we see the potential for human rights analysis to refine the Bath protocol.

One of the many tragic facts about COVID-19 is that epidemiologically relevant determinants of survival rates often coincide at the population level with traits that are ‘protected characteristics’ under human rights standards, including age and disability. COVID-19 triage procedures must accordingly walk something of a tightrope: incorporating sensitive epidemiological data into clinical decision-making without lapsing into discrimination. Citing guidance from the BMA, Cook and colleagues repudiate critical care ‘cut-off’ policies based on age or disability, on the grounds that they amount to unlawful direct discrimination. We concur. And we applaud the central pillar of their proposed alternative, which emphasises that triage decisions must always be made on an individual basis, taking into account individual circumstances and leaving scope for clinical judgement. Age will certainly inform individualised assessments, but under the Bath protocol the ultimate driver would be *prognosis*—specifically prognosis for recovery with the help of emergency treatment.

But avoidance of across-the-board age-based cut-offs does not suffice to safeguard against age discrimination. A triage policy built on the foundation of human rights must also be alive to the possibility of indirect discrimination. The Bath protocol’s incorporation of a life-years approach certainly raises the spectre of indirect discrimination, as its implementation would result in triage decisions that particularly disadvantage older persons. This by itself does not show that a life-years approach is unacceptable, but it does mean that such an approach calls for strict scrutiny. Specifically, it would need to be shown that there exists an ‘objective and reasonable justification’ for a life-years approach. Does it pursue a legitimate aim, and are the chosen means proportionate to that aim?^7^

We are doubtful about the ability of the life-years component of the Bath protocol to survive strict scrutiny. Cook and colleagues have not themselves specified how the second conjunct of their prime imperative would work in practice. Consider two possibilities. A life-years criterion might contribute to triage decisions as a straightforward function of age, with younger people assigned a higher life-years rating than their elders. But such an approach would be functionally indistinguishable from an age cut-off, which the Bath protocol rightly renounces. It would also not constitute an objective assessment of prospective life-years since life expectancy varies by factors other than age. An alternative more in keeping with the Bath approach would be to undertake an individualised assessment of expected life-years for each patient, taking into account the various demographic factors that influence life expectancy, including socioeconomic status and disability.^8^ The negative impacts of such an approach on already disadvantaged groups within society is difficult to gauge, but they are sufficiently grave to raise doubts as to whether such a policy could pass a proportionality test, which is satisfied only if it can be shown that the impact of the policy on the interests of those groups is not ‘excessive’ in relation to the aim being pursued.^9^

## Equality and equal worth

The overarching purpose of the modern human rights movement is to protect the equal dignity and worth of all human beings.^10^ In order to fulfil this purpose in the context of healthcare, international human rights standards call for individuals to be provided with equal opportunities to access healthcare services.^11^ This equal access imperative is in turn echoed in the NHS Constitution.^12^ The principle that all lives are of equal dignity and worth provides additional grounds for scepticism about a life-years approach to triage, which implicitly treats a longer life as intrinsically more worth saving than a shorter one. But its importance as a supplement to the ethical framework of the Bath protocol becomes particularly clear in the context of so-called ‘tie-breakers.’

Prognosis-driven triage reaches an intrinsic limit when clinical factors do not suffice to distinguish among patients. At that point, some supplementary principle of distinction must be adopted. The most prominent tie-breaking recommendation within the Bath proposal is that a ‘random allocation, such as a lottery’ be used to rank patients with similar prognoses. But the protocol also invokes two more controversial tie-breaking mechanisms: priority for individuals with the potential to contribute to the maintenance of critical infrastructure; and priority for volunteers in vaccine trials and other medical research relevant to the pandemic. These additional tie-breaking standards have an equivocal status within the protocol; the authors describe them as “retained but downgraded.”

The prioritisation of patients based on ‘factors that might benefit wider society’ clearly invites scrutiny based on the principle of equality. For every patient moved up in a triage queue on the basis of their social utility, someone else is moved down, limiting their access to healthcare services on the basis of an assessment of their lower predicted value to society.

How do the two ‘downgraded’ tie-break mechanisms fare when assessed in a human rights framework? Consider first a policy of ‘rewarding’ volunteers in vaccine trials. Suppose that the aim of such a policy is to ensure that there is a sufficient supply of participants in these vitally important trials, and more broadly to ensure that the trials can be successfully completed. This would certainly pass the ‘legitimate aim’ arm of the test. But in the absence of some compelling link between such a system of rewards and the success of the trials programme, it would fail to satisfy the proportionality test and so fail to constitute a permissible departure from the duty to promote and respect equality—a duty which stands at the head of the NHS Constitution. Otherwise put: individuals have a right to be treated equally in triage regardless of whether they volunteered for a vaccine trial, chose not to, or were never presented with the opportunity in the first place.

Applying the same standard of scrutiny to priority for ‘front line workers’ is more challenging. Ensuring the continuing operation of critical infrastructure certainly passes the legitimate aim test. Where staff shortages are sufficiently acute, prioritisation of care for frontline workers might become a necessary means for protecting the workforce. But prioritisation on the basis of ‘social utility’ still faces two acute challenges: objectively identifying the point at which infrastructural strains justify prioritisation and providing a reasonable definition of ‘frontline worker’ for those purposes. Because these challenges are not addressed in the Bath protocol, it does not provide an adequate justification for using such a principle of prioritisation.

## Conclusion

We are not under the illusion that a human rights framework will provide definitive answers to the many challenges associated with triage policy. We submit, however, that human rights considerations must have a central place in the ‘ethical context in a pandemic,’ and that their inclusion provides a set of analytical tools to help structure the assessment of triage proposals. The principle of ‘saving more lives’ is a classic example of a consequentialist ethical framework. That is, it defines the ethical context of triage by defining the desired outcome. An approach to triage informed by human rights standards will recognise that there are ethically significant constraints on the means by which such an aim is to be achieved. We have argued here that these constraints include a commitment to avoid direct discrimination and also a practice of ‘strict scrutiny’ for policies that threaten indirect discrimination, together with robust commitment to the principle that all human lives have equal worth and dignity. These principles, supplemented by the rigorous application of the established human rights tests for necessity and proportionality, can play a role in shaping triage practices that meet the highest possible ethical standards.
